# Effects of progranulin on the pathological conditions in experimental myocardial infarction model

**DOI:** 10.1038/s41598-020-68804-7

**Published:** 2020-07-16

**Authors:** Takahiro Sasaki, Masamitsu Shimazawa, Hiromitsu Kanamori, Yoshihisa Yamada, Anri Nishinaka, Yoshiki Kuse, Genjiro Suzuki, Tomomi Masuda, Shinsuke Nakamura, Masato Hosokawa, Shinya Minatoguchi, Hideaki Hara

**Affiliations:** 1 Molecular Pharmacology, Department of Biofunctional Evaluation, Gifu Pharmaceutical University, 1-25-4 Daigaku-nishi, Gifu, 501-1196 Japan; 2 Department of Cardiology, Gifu University Graduate School of Medicine, Gifu, Japan; 3 Dementia Research Project, Department of Dementia and Higher Brain Function, Tokyo Metropolitan Institute of Medical Science, Tokyo, Japan; 4 Department of Circulatory and Respiratory Advanced Medicine, Gifu University Graduate School of Medicine, Gifu, Japan; 5 Heart Failure Center, Gifu Municipal Hospital, Gifu, Japan

**Keywords:** Drug discovery, Cardiology

## Abstract

Progranulin is a secreted growth factor associated with multiple physiological functions in ischemic pathophysiology. However, it is still not fully understood how progranulin is involved in ischemic lesion and cardiac remodeling after myocardial infarction (MI). In this study, we investigated the effects of progranulin on myocardial ischemia and reperfusion injury. We investigated progranulin expression using Western blotting and immunostaining after permanent left coronary artery (LCA) occlusion in mice. Infarct size and the number of infiltrating neutrophils were measured 24 h after permanent LCA occlusion. Recombinant mouse progranulin was administered before LCA occlusion. In addition, we evaluated cardiac function using cardiac catheterization and echocardiography, and fibrosis size by Masson’s trichrome staining after myocardial ischemia/reperfusion in rabbits. Recombinant human progranulin was administered immediately after induction of reperfusion. Progranulin expression increased in the myocardial ischemic area 1, 3, and 5 days after permanent LCA occlusion in mice. The administration of recombinant mouse progranulin significantly attenuated infarct size and infiltrating neutrophils 24 h after permanent LCA occlusion in mice. We also found that administration of recombinant human progranulin ameliorated the deterioration of cardiac dysfunction and fibrosis after myocardial ischemia/reperfusion in rabbits. These findings suggest that progranulin may protect myocardial ischemia/reperfusion injury.

## Introduction

Cardiovascular diseases (CVD) are disorders of the heart and blood vessels, and a leading cause of death worldwide despite therapeutic intervention ^1^. The worldwide prevalence of CVD is approximately 17.7 million people every year, and CVD accounts for 30% of global mortality ^2^. Acute myocardial infarction (AMI) in CVD leads to the sudden cardiac death and heart failure, which is a devastating complication ^3^. AMI is an event of myocardial necrosis by acute thrombotic obstructions of blood flow in coronary arteries. Rapid reperfusion of coronary arteries achieved by percutaneous coronary intervention (PCI) and thrombolysis benefits patients with AMI ^4,5^. However, there are several problems with current therapy. Myocardial ischemia/reperfusion (I/R) injury increases myocardial infarct size and decreases blood flow associated with microcirculatory disturbances ^6–8^. Enlargement of I/R injury from delayed reperfusion therapy increases the risk of subsequent developments of cardiac rupture and heart failure, in patients with AMI ^7^. In addition, these therapies and subsequent therapeutic interventions increase the mental and economic burden on patients ^9,10^. Therefore, it is important to elucidate the pathogenesis of myocardial I/R injury and explore novel therapeutic targets for AMI.

Progranulin is a secreted growth factor associated with embryonic development ^11^, wound healing ^12^, and inflammation ^13,14^. Progranulin expression is observed in macrophages, neutrophils and skeletal myocytes ^15,16^. Progranulin gene mutations have been identified in the pathogenesis of frontotemporal lobar degeneration associated with the accumulation of TAR DNA-binding protein 43 (TDP-43) ^17^. Previous reports have shown that progranulin protects against I/R injury in the heart, brain and kidney ^18–20^. In our previous report, the administration of recombinant progranulin also attenuated neuronal injury by inhibiting neutrophil recruitment in a focal cerebral I/R injury murine model ^19^. It recently has been reported that progranulin protects cardiac dysfunction in the early phase after myocardial I/R injury ^18^. However, it is still not fully understood how progranulin is involved in ischemic lesion and cardiac remodeling after AMI. In this study, we investigated the effects of progranulin on ischemic lesion and cardiac remodeling after myocardial I/R and permanent ischemia using experimental animal models of MI.

## Results

We used 22 mice in vehicle-treated group, and 17 mice in progranulin-treated group in the experiments to investigate the effects of recombinant progranulin on MI. In the experiments, 10 mice in vehicle-treated group and 2 mice in progranulin-treated group died within 1 day after permanent occlusion of LCA. Survival rate was 55% (n = 12/22) in vehicle-treated group, and 89% (n = 15/17) in progranulin-treated group within 1 day after permanent occlusion MI.

### Expression of progranulin in the heart after permanent left coronary artery occlusion in mice

**Figure 1:**
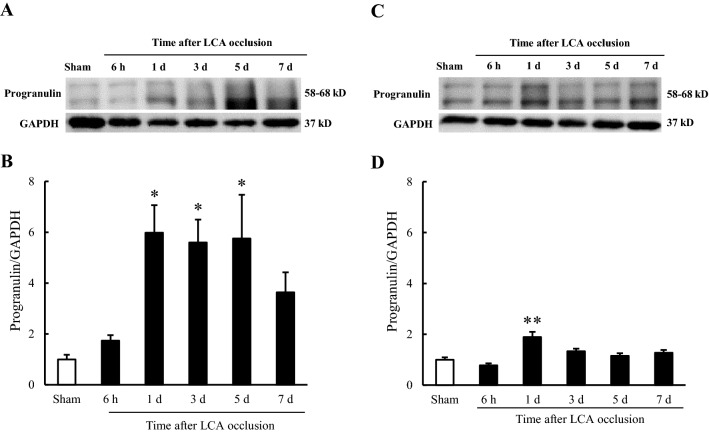
Progranulin expression in the heart after myocardial infarction in mice. (**A**) Representative images show progranulin (58–68 kD) in the ischemic area 6 h and 1, 3, 5, and 7 days after permanent occlusion of left coronary artery (LCA) by Western blotting. (**B**) Quantitative analysis of progranulin normalized to GAPDH in the ischemic area. (**C**) Representative images show progranulin (58–68 kD) in non-ischemic area 6 h and 1, 3, 5, and 7 days after permanent occlusion of LCA by Western blotting. (**D**) Quantitative analysis of progranulin normalized to GAPDH in non-ischemic area. The expression of progranulin increased in the ischemic area 1, 3, and 5 days after permanent occlusion of LCA. Data are the means ± SEM. (n = 5–7) ^*^p < 0.05, ^**^p < 0.01 versus sham-operated group (one-way ANOVA followed by Dunnett's test).

We investigated the expression of progranulin in ischemic and non-ischemic myocardium 6 h and 1, 3, 5, and 7 days after permanent left coronary artery (LCA) occlusion in mice. In the ischemic areas, progranulin expression was significantly increased 1, 3, and 5 days after permanent occlusion MI (Fig. 1A, B, Supplementary Figure S1A). On the other hand, the expression of progranulin was increased only 1 day after permanent occlusion of the LCA in non-ischemic areas (Fig. 1C, D, Supplementary Figure 1B).

### Localization of upregulated progranulin and exploration of progranulin expressed cells

**Figure 2:**
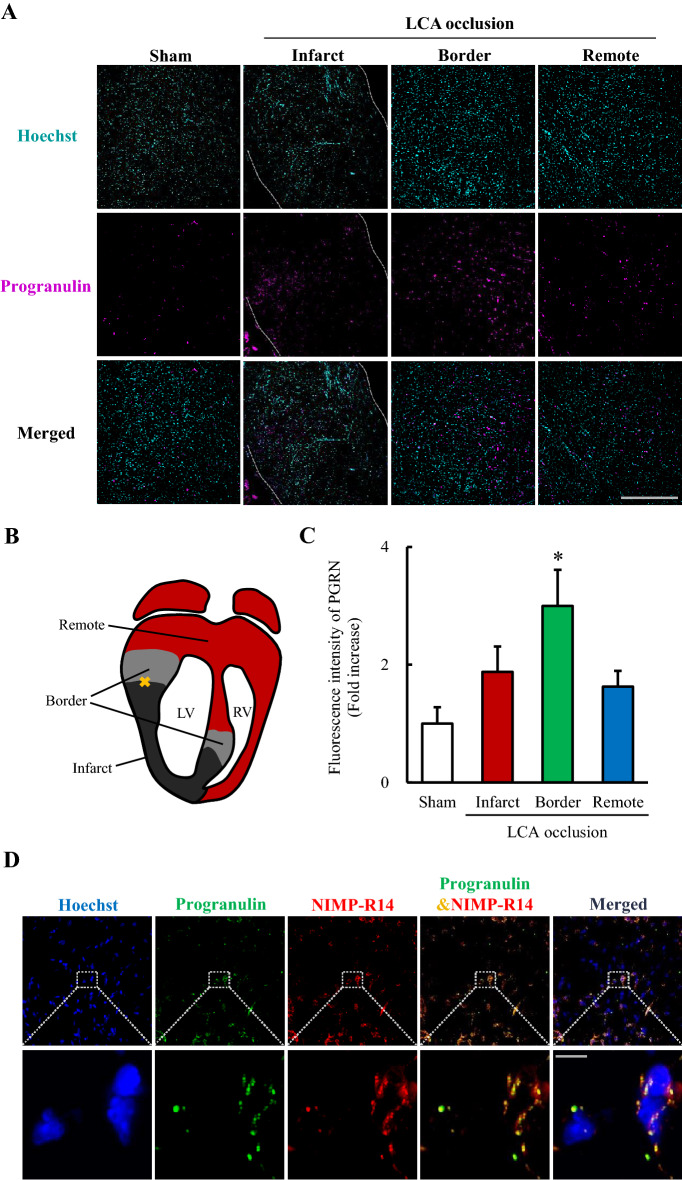
Localization of upregulated progranulin and exploration of progranulin expressed cells after myocardial infarction in mice. (**A**) Representative images show immunostaining for progranulin in sham-operated group, and at infarct, border and remote areas 1 day after permanent occlusion of LCA. Nuclei were stained with Hoechst 33342. Scale bar: 500 μm. (**B**) Scheme of heart section indicating the infarct, border, and remote areas after permanent occlusion of LCA. (**C**) Quantitative analysis of fluorescence intensity of progranulin. Progranulin expression significantly increased in the border area 1 day after permanent occlusion of LCA. (**D**) Representative images at ×60 and ×600 magnification show immunostaining for progranulin and NIMP-R14 at the border area 1 day after permanent occlusion of LCA. Scale bar at the ×60 and ×600 magnification: 50 μm and 5 μm representatively. Data are the means ± SEM. (n = 5–6) ^*^p < 0.05 versus sham-operated group (one-way ANOVA followed by Dunnett's test).

We investigated the localization of upregulated progranulin, and the identification of progranulin expressed cells 1 day after permanent occlusion of LCA using immunostaining. We evaluated progranulin expression levels at the infarct, border, and remote areas as shown in scheme of heart section (Fig. 2B). Progranulin expression significantly increased at the border area 1 day after permanent occlusion MI (Fig. 2A, C). Progranulin was merged with neutrophils marker NIMP-R14 by immunostaining (Fig. 2D).

### Administration of recombinant progranulin decreased infarct size after myocardial infarction in mice

**Figure 3:**
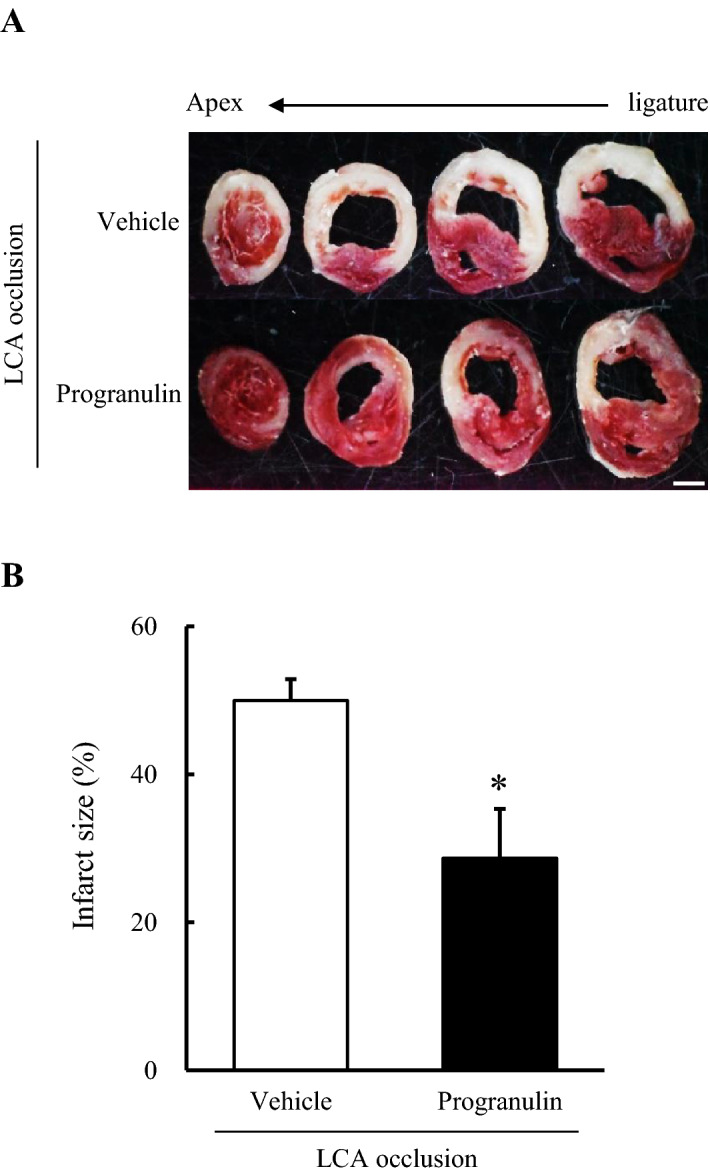
Administration of recombinant progranulin decreased infarct size after myocardial infarction in mice. (**A**) Representative photographs show the heart sections stained by 2, 3, 5-Triphenyl tetrazolium chloride (TTC) solution, 24 h after permanent occlusion of LCA. (**B**) Quantitative analysis of infarct size 24 h after permanent occlusion of LCA. The administration of recombinant progranulin significantly decreased myocardial infarct size compared with vehicle-treated group. Data are the means ± SEM. (n = 7–9) ^*^p < 0.05 vs. vehicle-treated group (two-tailed Student's *t*-test).

We investigated whether recombinant progranulin decreased infarct size 24 h after permanent occlusion of LCA. Intravenous administration of recombinant mouse progranulin (300 μg/kg) immediately before ligation of LCA significantly reduced myocardial infarct size by 21% compared with vehicle-treated group 24 h after permanent occlusion of the LCA (Fig. 3A, B).

### Administration of recombinant progranulin suppressed the infiltrating neutrophils after myocardial infarction in mice

**Figure 4:**
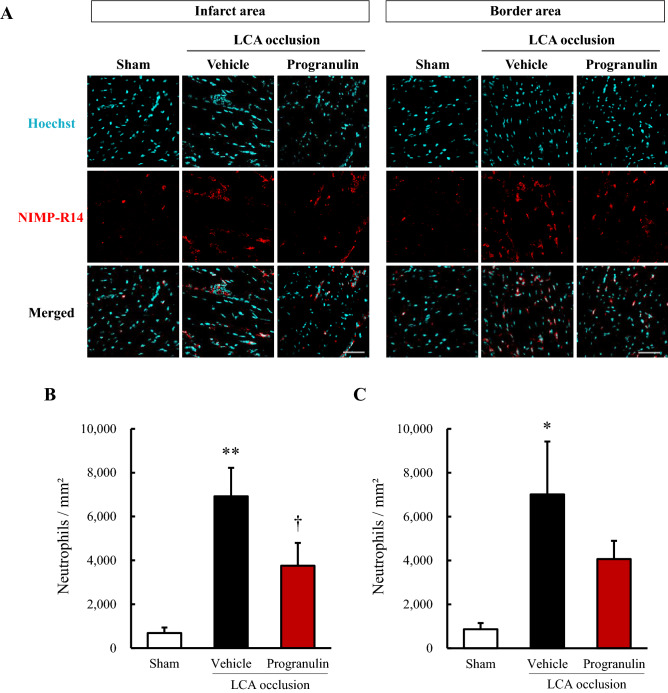
Administration of recombinant progranulin suppressed the infiltrating neutrophils after myocardial infarction in mice. (**A**) Reparative images show immunostaining for NIMP-R14 at the infarct and border areas 1 day after permanent occlusion of LCA. Nuclei were stained with Hoechst 33342. (**B**) Number of neutrophils per mm^2^ at infarct area after permanent occlusion of LCA. (**C**) Number of neutrophils per mm^2^ at border area after permanent occlusion of LCA. The administration of recombinant progranulin reduced the number of infiltrating neutrophils at the infarct area compared with vehicle-treated group. Scale bar: 50 μm. Data are the means ± SEM. (n = 5–6) ^*^p < 0.05, ^**^p < 0.01 versus sham-operated group (two-tailed Student's *t*-test), ^†^*p* < 0.05 vs. vehicle-treated group (one-tailed Student's *t*-test).

We investigated whether recombinant progranulin suppressed the infiltration of neutrophils 1 day after permanent occlusion of LCA. The number of neutrophils significantly decreased at infarct area and was tendency to decrease at border area in recombinant mouse progranulin-treated group compared with vehicle-treated group (Fig. 4A–C).

### Administration of recombinant progranulin decreased deterioration of cardiac dysfunction after myocardial ischemia–reperfusion injury in rabbits

First, we investigated the property and effects of recombinant human progranulin prepared as described previously^21^. Two bands (58–68 kDa) were detected in the culture medium from SH-SY5Y transfected with pcDNA3.1 ( +)-PGRN using CBB assay (Supplementary Figure S2A). The bands were detected by western blotting for progranulin (Supplementary Figure S2B). The cell viability in H9c2 and SH-SY5Y cells significantly increased in the culture medium from pcDNA3.1 ( +)-PGRN treated-group compared with PBS and culture medium from SH-SY5Y transfected with pcDNA3.1 (Supplementary Figure 2C, D). It was no significant difference of cell viability in both cells between the culture medium from SH-SY5Y transfected with pcDNA3.1 treated-group and PBS-treated group (Supplementary Figure 2C, D). Thus, the culture medium from SH-SY5Y transfected with pcDNA3.1 ( +)-PGRN was hereinafter referred to as recombinant human progranulin.

**Figure 5:**
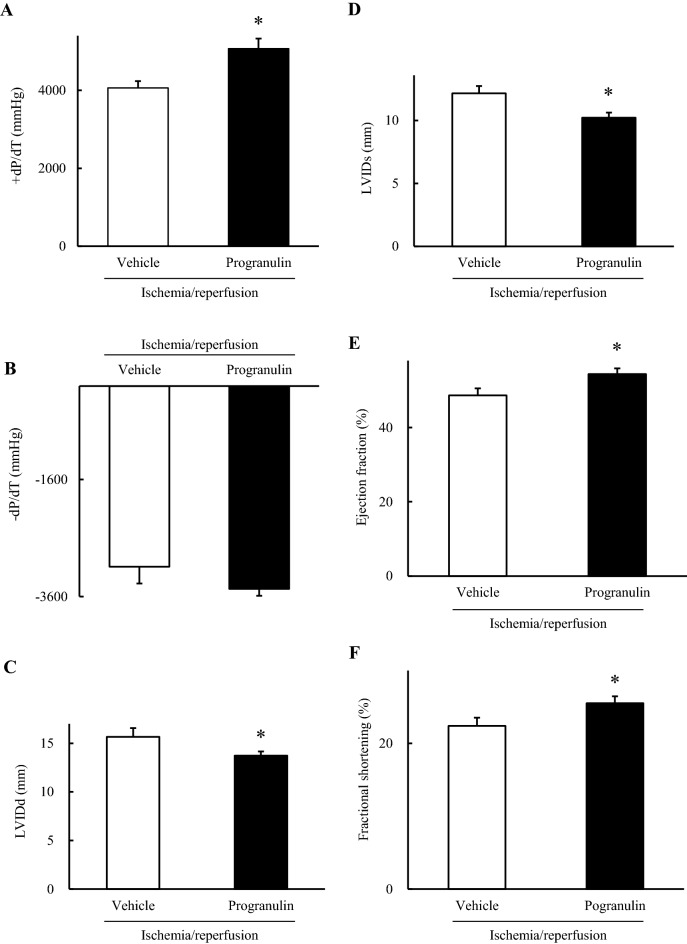
Administration of recombinant progranulin decreased deterioration of cardiac dysfunction after myocardial ischemia–reperfusion injury in rabbits. (**A**) The positive values of the first derivative in left ventricular pressure (dP/dt) and (**B**) the negative dP/dt were determined by cardiac catheterization 2 weeks after myocardial ischemia/reperfusion (I/R). (**C**) Left ventricular internal diameter at diastole (LVIDd), (**D**) left ventricular internal diameter at systole (LVIDs), (**E**) ejection fraction (EF), and (**F**) fractional shortening (FS) were measured by echocardiography analysis. The administration of recombinant progranulin significantly ameliorated the deterioration of + dP/dt, LVIDd, LVIDs, EF and FS compared with vehicle-treated group. Data are the means ± SEM. (n = 3–7) ^*^p < 0.05 versus vehicle-treated group (**A**, **B**; two-tailed Student's *t*-test, **C**–**F**; one-tailed Student's *t*-test).

We investigated the effects of recombinant progranulin against the cardiac function after myocardial I/R in rabbits. It was no significant difference of cardiac function between vehicle-treated group and recombinant human progranulin-treated group, before induction of myocardial I/R injury (Supplementary Figure S3A-D). The first derivative of the left ventricular pressure with time (dP/dt) is related to cardiac contractility ^22^. In cardiac catheter analysis, positive dP/dt significantly improved in the recombinant human progranulin-treated group compared with the vehicle-treated group 2 weeks after myocardial I/R (Fig. 5A). On the other hand, negative dP/dt in the recombinant human progranulin-treated group was not significantly different from the vehicle-treated group after myocardial I/R (Fig. 5B). LV internal diameter is associated with an index of adverse cardiac remodeling. The administration of recombinant human progranulin significantly attenuated the deterioration of LV internal diameter at systole (LVIDs) and diastole (LVIDd) in the echocardiography analysis (Fig. 5C, D). The administration of recombinant progranulin also significantly improved LV ejection fraction (EF) and fractional shortening (FS) compared with vehicle-treated group (Fig. 5E, F).

### Administration of recombinant progranulin decreased myocardial fibrosis size after myocardial ischemia–reperfusion in rabbits

**Figure 6:**
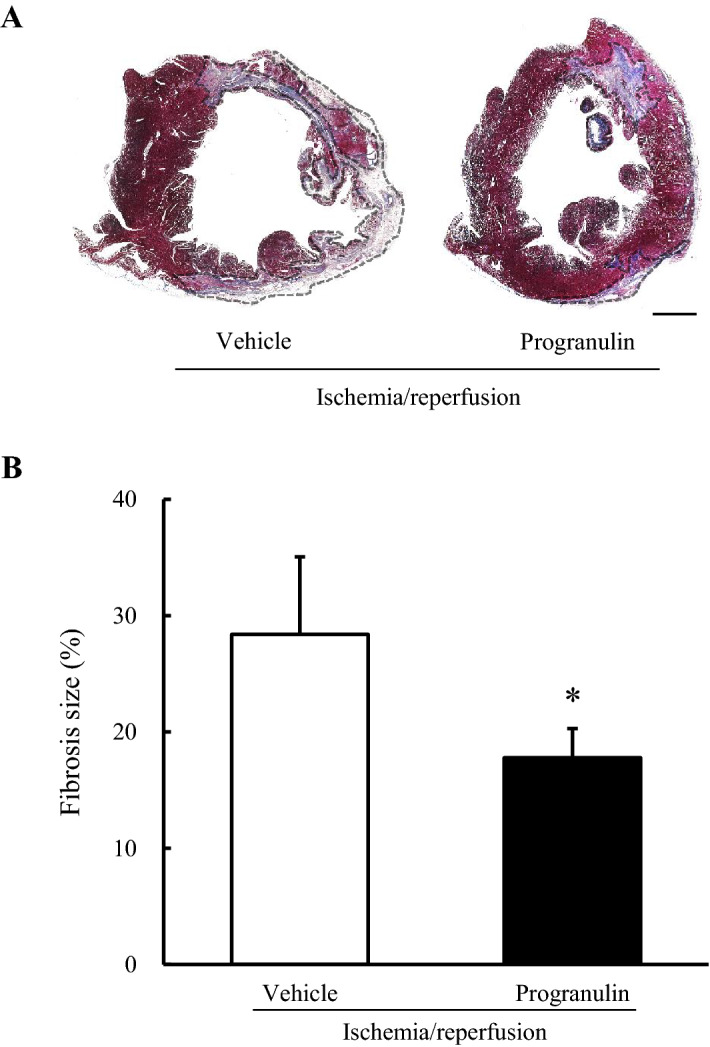
Administration of recombinant progranulin decreased myocardial fibrosis size after myocardial ischemia–reperfusion in rabbits. (**A**) Representative photographs show LV cross-sections stained by Masson’s trichrome solutions 2 weeks after myocardial I/R. (**B**) Quantitative analysis of fibrosis size 2 weeks after myocardial I/R. The administration of recombinant progranulin significantly reduced fibrosis size compared with vehicle-treated group. Scale bar: 2.5 mm. Data are the means ± SEM. (n = 3–7) ^*^p < 0.05 versus vehicle-treated group (one-tailed Student's *t*-test).

A fibrotic scar is mainly formed in the myocardial region subjected to ischemia and reperfusion injury after MI to prevent ventricular wall rupture ^23,24^. However, previous reports indicated that fibrosis increases ventricular stiffness, which is associated with cardiac contractility modulation and congestive heart failure ^25^. Therefore, we investigated whether the recombinant progranulin decreased myocardial fibrosis 2 weeks after I/R in rabbits using Masson’s trichrome staining. The administration of recombinant human progranulin significantly decreased fibrosis size in myocardium by 10% compared with the vehicle-treated group after myocardial I/R. (Fig. 6A, B).

## Discussion

In the present study, we revealed that administration of recombinant progranulin decreased infarct size and infiltrating neutrophils after permanent LCA occlusion in mice, and also improved cardiac dysfunction and fibrosis after myocardial I/R in rabbits.

Progranulin is a secreted growth factor associated with attenuation of inflammation after tissue injury ^19,26^. We found that the expression of progranulin significantly increased in the myocardial ischemic area, particularly the border area after permanent occlusion MI. Cardiomyocyte death in MI increases inflammatory factors and leukocyte infiltration, which promotes phagocytosis to remove dead cells and matrix debris ^27,28^. In the previous report, infarct size and subsequent cardiac dysfunction after MI accelerated by the increase of inflammatory factors from 6 to 24 h after permanent LCA occlusion in a murine model ^28,29^. On the other hand, progranulin expression significantly increased 24 h after focal cerebral I/R ^30^. It also has been reported that progranulin deficiency exacerbated the tissue injury through the increase of infiltrating neutrophils and macrophages after renal I/R injury in a murine model ^20^. Thus, we assumed that upregulated progranulin might be associated with cardiac protection through regulating the inflammation after MI.

Progranulin expression was upregulated in immune cells including neutrophils and macrophages during wound healing and ischemic conditions ^12,30^. We found that progranulin was merged with neutrophils marker NIMP-R14 after induction of permanent occlusion MI. It suggests that neutrophils might be one of the cells which expressed progranulin after MI. Neutrophils infiltrate the infarct area in the first few hours following onset of myocardial ischemia. They produce reactive oxygen species and granule components including myeloperoxidase, thereby exacerbating tissue injury ^31^. On the other hand, a recent study has reported that infiltration of neutrophils were required to resolve inflammation post-MI ^32^. The previous reports implicate that neutrophils associate with both pro-inflammation and tissue repair after MI. We also previously reported that progranulin suppressed the migration of neutrophils ^19^. Therefore, progranulin secreted from neutrophils might regulate inflammation after MI by autocrine mechanism and effect on other immune cells.

We found that the intravenous administration of recombinant progranulin significantly decreased infarct size 24 h after permanent LCA occlusion in mice. We also found that recombinant progranulin significantly suppressed infiltrating neutrophils at infarct area 1 day after permanent occlusion of LCA. In our previous report, recombinant progranulin significantly attenuated the neuronal injury after focal cerebral I/R by inhibition of recruited neutrophils ^19^. It also has been reported that recombinant progranulin significantly attenuated the infiltrating neutrophils after renal I/R injury ^20^. The previous reports implicate that progranulin reduces the tissue injury in ischemia and reperfusion by inhibition of infiltrating neutrophils. A recent study have shown that excessive accumulation of neutrophils exacerbated myocardial infarct size 24 h after permanent occlusion of LCA ^33^. In addition, attenuating the recruitment of neutrophils decreased the infarct size in permanent occlusion of LCA and myocardial I/R injury ^31,34^. It is implicated that inhibition of neutrophil activation attenuates tissue injury after MI. Thus, our findings suggest that recombinant progranulin might decrease infarct size after permanent occlusion of LCA, at least in part, through suppression of infiltrating neutrophils.

Formation of cardiac fibrosis leads to the maintenance of tissue integrity in reparative response after MI ^27^. However, tissue sclerosis provoked by fibrosis is associated with the impairment of cardiac contractility ^25^. Deterioration of fibrosis in myocardium is related to cardiac dysfunction after MI ^35,36^. It has been reported that serum levels of progranulin is associated with the degree of hepatic fibrosis in patients with nonalcoholic fatty liver disease ^37^. Progranulin also attenuated liver fibrosis after chronic liver injury in mice by downregulating the inflammatory response ^38^. It is implicated that progranulin is associated with the tissue fibrosis in inflammatory conditions. We found that administration of recombinant progranulin ameliorated the deterioration of LV remodeling and cardiac dysfunction after myocardial I/R in rabbits. In addition, fibrosis size in myocardium was significantly decreased by administration of recombinant progranulin. Initial injury provoked by MI associates with subsequent cardiac dysfunction and high mortality ^39,40^. A recent study has reported that administration of recombinant human progranulin significantly improved cardiac function through activation of PI3K/Akt signaling pathway, 30 min after ischemia followed by 60 min reperfusion in rats ^18^. Therefore, in our study, protective effects of progranulin on myocardial I/R injury might be associated with initial activation of PI3K/Akt. Otherwise, Wnt signaling has a crucial role in fibrosis formation and cardiac function after MI ^41,42^. Upregulation of Wnt signaling accelerates the cardiac injury and adverse cardiac remodeling after MI ^43,44^. It has been reported that the inhibition of Wnt signaling suppressed the cardiac remodeling and fibrosis after MI in mice ^44^. On the other hand, progranulin has been reported to regulate Wnt signaling by inhibiting Wnt protein expression in inflammatory pathophysiology ^45^. In a previous report, progranulin also ameliorated the hyperhomocysteinemia-induced cardiorenal injury by inhibiting Wnt signaling ^46^. Therefore, it is implicated that administration of recombinant progranulin might ameliorate the deterioration of cardiac dysfunction and fibrosis by inhibiting Wnt signaling after MI.

In our study, we investigated the effects of progranulin on MI using permanent occlusion of LCA in mice and myocardial I/R in rabbits. It is a difference of pathophysiology between permanent ischemia and I/R in myocardium ^1,6^. Cardiomyocyte death was mainly caused by prolonged deprivation of oxygen and nutrient supply in myocardial ischemia. On the other hand, abrupt oxidative stress provoked by reoxygenation exacerbates tissue injury in myocardium after reperfusion. However, both pathophysiological conditions cause extensive tissue injury associated with the inflammatory response at ischemic and non-ischemic areas after MI. Indeed, neutrophils and inflammatory cytokines significantly increased in the heart at 1 day after permanent occlusion of LCA and myocardial I/R ^47–49^. In the previous reports, suppression of the inflammatory response including infiltrating neutrophils decreased myocardial infarct size and improved cardiac function in permanent occlusion of LCA ^50,51^ and myocardial I/R ^52,53^ . Progranulin has been reported to suppress the inflammatory response associated with functional modulation of neutrophils and regulatory T cells via binding to TNF receptor ^19,54^. Therefore, progranulin might protect against myocardial ischemia and reperfusion injury, at least in part, through regulating inflammatory response after MI.

We performed the administration of recombinant progranulin before occlusion of LCA in mice. The time of administration limits the clinical implications of our findings. However, the findings of this study suggest that upregulation of progranulin expression after induction of MI may be associated with protective roles against the myocardial ischemic injury. It is important to understand the role of progranulin in myocardial ischemic conditions.

In conclusion, we demonstrated that the dynamic changes of expression and localization of progranulin after MI. Progranulin may have therapeutic potential for myocardial ischemia and reperfusion injury.

## Methods and materials

### Animals

Male ICR mice (8–10 weeks old) and Japanese White rabbits (10 weeks old) were purchased from Japan SLC Ltd (Shizuoka, Japan). Animal protocols were performed in accordance with the National Institutes of Health Guidelines on the Use of Laboratory Animals and were approved and monitored by the Institutional Animal Care and Use Committee of Gifu Pharmaceutical University and Gifu University.

Animals in our animal facilities were maintained at 23 ± 3 °C under 12 h light–dark cycles with free access to food and water.

### Permanent left coronary artery occlusion in mice

Permanent occlusion of left coronary artery (LCA) in mice was performed as described previously ^55–57^. Mice were anesthetized with 2.0–3.0% isoflurane and maintained with 1.0–1.5% isoflurane in 70% N_2_O and 30% O_2_. Anesthesia was delivered through a face mask with an animal general anesthesia machine (Soft Lander; Sin-ei Industry Co., Ltd., Saitama, Japan). A 20-gauge plastic cannula was intubated into the trachea and connected to a rodent ventilator (Model 687; Harvard Apparatus, Massachusetts, USA) via a plastic tube. Left thoracotomy was performed to visualize the left auricle. LCA was ligated using a 7–0 silk suture. LCA occlusion was confirmed by regional cyanosis of the myocardial surface. The muscle layers and skin were closed using a 3–0 suture. Recombinant mouse progranulin (300 µg/kg, R&D systems, Inc., Minneapolis, MN, USA) was injected into a tail vein just before occlusion of LCA. The same volume of PBS (10 mL/kg) was intravenously injected for the vehicle group. In sham-operated mice, the same surgical procedures were performed without ligation of LCA. Mice body temperature was maintained at 37 °C with a heat lamp during the procedures.

### Measurement of infarct size in mice

Mice were euthanized with an intraperitoneal injection of sodium pentobarbital (50 mg/kg i.p.) 24 h after permanent occlusion of LCA. Isolated heart was sliced transversely into 4 sections below the ligation site. The sections were incubated with 2% TTC in saline for 20 min at room temperature and fixed in 10% buffer formalin overnight. The slices were weighted and imaged with a digital camera (Coolpix 4,500; Nikon, Tokyo, Japan). Infarct area was measured using image-processing software (ImageJ ver. 1.52v; National Institutes of Health, Bethesda MD, USA). The infarct size was calculated as percentage of infarct volume in the total left ventricular (LV) volume.

### Myocardial ischemia–reperfusion in rabbits

Myocardial ischemia/reperfusion (I/R) in rabbit was performed as described previously ^56–58^. The rabbits were anesthetized with an intravenous injection of sodium pentobarbital (30–40 mg/kg). Orotracheal intubation was performed using uncuffed endotracheal tube. The rabbits were ventilated with room air supplemented using a mechanical ventilator (tidal volume, 25–35 mL, respiratory rate: 20–30 breaths/min, Model SN-480-5; Shimano, Tokyo, Japan). The skin was incised and the intercoastal muscles were transected after intramuscular injection of 1% lidocaine hydrochloride. The pericardium is dissected to expose LCA. A 4–0 silk thread was passed around a prominent branch of the coronary artery. Regional ischemia is induced by pulling the ends of the suture through a small polyethylene tube to form a snare. The successful induction of ischemia was verified by visual inspection (cyanosis). Reperfusion was performed by unclamping the tube. Successfully reperfused myocardium was distinguished by the recovery of the color on the ventricular surface. Recombinant human progranulin (300 µg/kg) was administrated through an ear vein immediately after reperfusion. The same volume of PBS (10 mL/kg) was intravenously injected for the vehicle group. The muscle layers and skin were closed using a 1–0 suture.

### Measurement of fibrosis area size in rabbits

Rabbits were euthanized by an intravenous injection of sodium pentobarbital (60–70 mg/kg i.v.) following 30 min ischemia and 2 weeks reperfusion. The heart was isolated and perfused with 4% paraformaldehyde (PFA). The LV was weighed and sectioned transversely into 4 slices in the ischemic region. The section at the level of papillary muscle was fixed with 10% neutral buffered formalin overnight and embedded in paraffin. Six embedded-paraffin sections (thickness: 6 μm) were prepared and stained by Masson’s trichrome stain kit (Sigma-Aldrich, Inc., St. Louis, MO, USA). All images were digitally photographed using a fluorescence microscope (BZ-X710; Keyence, Osaka, Japan). The blue area stained by Masson’s trichrome kit were measured as fibrosis area using ImageJ. Fibrosis size was calculated as percentage of fibrosis area in the LV area, and shown as the average of three section images.

### Cardiac function analysis in rabbits

Cardiac function was analyzed by echocardiography and a catheter under the anesthesia as previously described ^58,59^. Echocardiography was performed at 2 weeks post-MI using an echocardiographic system (SSD2000; Aloka, Mitaka, Tokyo, Japan) equipped with a 3.8–7.5 MHz imaging transducer. Ejection fraction, fractional shortening, and LV end-diastolic and LV end-systolic dimensions were measured after intravenous injection of pentobarbital sodium (25 mg/kg). After echocardiography, a micromanometer-tipped catheter (SPR 407; Millar Instruments, Burnaby, BC, Canada) was inserted into the LV to record ± dP/dt.

### Western blot analysis

Mice were euthanized with an intraperitoneal injection of sodium pentobarbital (50 mg/kg i.p.). The heart was isolated and dissected into the ischemic core and non-ischemic remote area by distinguishing the color differences on the LV surface. The tissue was homogenized in lysis buffer (50 mM Tris HCl (pH 8.0), 150 mM NaCl, 0.5% sodium deoxycholate, 0.1% SDS, 1% IGEPAL CA-630, 1% Triton X-100, phosphatase inhibitor cocktail, and protease inhibitor cocktail (Sigma-Aldrich, Inc.)) using a homogenizer. The homogenate solution was centrifuged at 10,000 rpm for 20 min at 4 °C, and the protein concentration of the supernatant was measured compared to bovine serum albumin with a bicinchoninic acid protein assay kit (Pierce Biotechnology, Inc, MA, USA). A mixture of equal parts protein and sample buffer with 10% 2-mercaptoethanol was separated on a 5–20% sodium dodecyl sulfate–polyacrylamide gel electrophoresis (SDS-PAGE) gradient gel (SuperSep Ace; Wako Pure Chemical Industries, Ltd., Osaka, Japan), and the proteins were transferred to polvinylidene difluoride membranes (Immobilon-P; Millipore Corporation, Billerica, MA, USA). Transfer was followed by washing with TBS (T-TBS: 10 mM tris, 40 mM tris hydrochloride, 15 mM NaCl) with 0.05% Tween-20 solution (TBS-T). The transferred membranes were blocked for 30 min at room temperature with Blocking One-P (Nacalai Tesque, Inc., Kyoto, Japan) and then incubated overnight at 4 °C with different primary antibodies. The following primary antibodies were used for immunoblotting: sheep anti-progranulin (1:1,000, R&D systems, Inc.) and rabbit anti-GAPDH (1:1,000, Cell Signaling Technology, Tokyo, Japan). The secondary antibodies were horseradish peroxidase (HRP)-conjugated goat anti-rabbit (1:1,000, Pierce Biotechnology, Inc.) and HRP-conjugated rabbit anti-sheep IgG (1:1,000, Pierce Biotechnology, Inc.). Immunoreactive bands were visualized by Immuno Star LD (Wako Pure Chemical Industries, Ltd.) and a LAS-4000 Luminescent Image Analyzer (Fuji Film Co. Ltd., Tokyo, Japan).

### Immunofluorescence staining

Mice were euthanized with an intraperitoneal injection of sodium pentobarbital (50 mg/kg, i.p.) 1 day after the permanent occlusion of LCA. The heart was isolated, fixed in 4% PFA for 24 h at 4 °C and immersed in 30% sucrose for 24 h. Then, the heart was embedded in optimal cutting temperature compound (Sakura Finetechnical Co., Ltd., Tokyo, Japan). Transverse cryosections of 10 µm thickness were prepared using a cryostat vibratome (Leica CM 1,850; Leica Microsystems, Buffalo Grove, IL, USA), and placed on glass slides (MAS COAT; Matsunami Glass Ind., Ltd., Osaka, Japan). The sections were blocked with 5% normal horse serum (Sigma-Aldrich, Inc.) in PBS for 1 h at room temperature, and then incubated with the primary antibody for PGRN (1:100, R&D systems, Inc.) and NIMP-R14 (1:100, Abcam, Eugene, OR, USA) overnight at 4 °C. After the incubation, the sections were washed three times with PBS for 10 min each time, and then incubated with the secondary antibody Alexa Fluor 546 donkey anti-rabbit IgG (1:1,000, Molecular Probes, Eugene, OR, USA) and Alexa Fluor 647 donkey anti-sheep IgG (1:1,000, Molecular Probes) for 1 h at room temperature. After three washes with PBS, the sections were incubated in Hoechst 33342 (1:1,000, Molecular Probes) for 10 min to stain nuclear. Finally, the sections were mounted using Vectashield fluorescent mounting medium (Vector Laboratory, Burlingame, CA, USA) and cover-slipped for microscopy. The sections were visualized and digitally photographed using a confocal microscope at ×10, ×60 and ×600 magnification (Fluoview FV-10; Olympus, Tokyo, Japan). The fluorescence intensity was measured using ImageJ and shown as the average of three section images. Infarct, border and remote areas were distinguished as previously described ^60^. The areas of LV wall thinning were evaluated as the infarct area, and the areas within approximately 2 mm from the edges of infarct areas were defined as the border areas. The non-ischemic areas apparently away from infarct areas were also evaluated as the remote area.

### Preparation of recombinant human progranulin

SH-SY5Y neuroblastoma cells were cultured in DMEM/F12 medium (Sigma-Aldrich, Inc.) supplemented with 10% (v/v) Fetal Bovine Serum (FBS), penicillin–streptomycin (Thermo Fisher Scientific, Carlsbad, CA, USA), and Non-Essential Amino Acids Solution (Thermo Fisher Scientific) and maintained at 37 °C in the presence of 5% (v/v) CO_2_. Cells were grown to 40–50% confluence and transfected with pcDNA3.1 and pcDNA3.1 ( +)-PGRN using X-tremeGENE 9 DNA Transfection Reagent (Roche Diagnostics GmbH, Mannheim, Germany) according to the manufacturer’s instructions ^21^. Two days after transfection, medium was changed to DMEM/F12 medium without FBS. One day after medium exchange, medium was collected and centrifuged to remove cells. The supernatant containing secreted progranulin was precipitated with 50% saturated ammonium sulfate. The precipitated pellet was suspended in 30 mM Tris–HCl, pH 7.5. The solution was hereinafter referred to as recombinant human progranulin. Protein concentration was determined by Protein Assay CBB Solution (Nacalai Tesque, Inc.).

### Cell culture

Rat cardiomyocytes (H9c2) (European Collection of Authenticated Cell Culture, Wiltshire, UK) and human neuroblastoma (SH-SY5Y) cells (European Collection of Cell Culture) were maintained in Dulbecco’s modified Eagle’s medium (DMEM) with high glucose (Sigma-Aldrich, Inc.) containing 100 units/mL penicillin, 100 µg/mL streptomycin (Meiji Co. Ltd., Tokyo, Japan) and 10% fetal bovine serum (FBS; Valeant, Costa Mesa, CA, USA) at 37 °C and 5% CO_2_.

### Cell proliferation assay

H9c2 and SH-SY5Y cells were seeded at 1 × 10^5^ cells/mL into 96 well plates with DMEM containing 10% FBS and cultured at 37 °C and 5% CO_2_ for 24 h. Medium was then replaced with DMEM without FBS. Cells were treated with the conditioned medium of SH-SY5Y transfected with pcDNA3.1 ( +)-progranulin or pcDNA3.1 for 24 h. Cell Counting Kit-8 (CCK-8) solution was added to the cell culture. The cells were incubated at 37 °C and 5% CO_2_ for 3 h, and the absorbance measured at 450 nm (reference wave length, 600 nm) using a spectrophotometer (Varioskan; Thermo Electron Corporation, Vantaa, Finland).

### Statistical analysis

Data were expressed as mean ± SEM. Quantitative variables were statistically analyzed using Student’s one- or two-tailed *t*-test and one-way ANOVA followed by Dunnett’s test. *P*-values of less than 0.05 were considered as statistically significant.

## Supplementary information

Supplementary file1 (PDF 350 kb)
