# Supplementary material for: Effects of progranulin on the pathological conditions in experimental myocardial infarction model
Source: Sci Rep. 2020 Jul 16;10:11842. doi: 10.1038/s41598-020-68804-7 (PMC7367277; doi:10.1038/s41598-020-68804-7)
Supplement: Supplementary file 1 — Supplementary file1 (PDF 350 kb) [file 41598_2020_68804_MOESM1_ESM.pdf]

Supplementary information

Effects of progranulin on the pathological conditions in experimental myocardial infarction model

Takahiro Sasaki[1], Masamitsu Shimazawa[1], Hiromitsu Kanamori[2], Yoshihisa Yamada[2], Anri Nishinaka[1], Yoshiki Kuse[1], Genjiro Suzuki[3], Tomomi Masuda[1], Shinsuke Nakamura[1], Masato Hosokawa[3], Shinya Minatoguchi[4, 5], and Hideaki Hara[1*]

Afilliations:

[1]Molecular Pharmacology, Department of Biofunctional Evaluation, Gifu Pharmaceutical University, Gifu, Japan.

[2] Department of Cardiology, Gifu University Graduate School of Medicine, Gifu, Japan.

[3] Dementia Research Project, Tokyo Metropolitan Institute of Medical Science, Tokyo, Japan.

[4] Department of Circulatory and Respiratory Advanced Medicine, Gifu University Graduate School of Medicine, Gifu, Japan.

[5] Heart Failure Center, Gifu Municipal Hospital, Gifu, Japan.

[*]Corresponding Author:

Professor Hideaki Hara, R.Ph., Ph.D.,

Molecular Pharmacology, Department of Biofunctional Evaluation, Gifu Pharmaceutical University, 1-25-4 Daigaku-nishi, Gifu 501-1196, Japan.

TEL/FAX: +81-58-230-8126

E-mail: hidehara@gifu-pu.ac.jp

Supplementary Figure 1

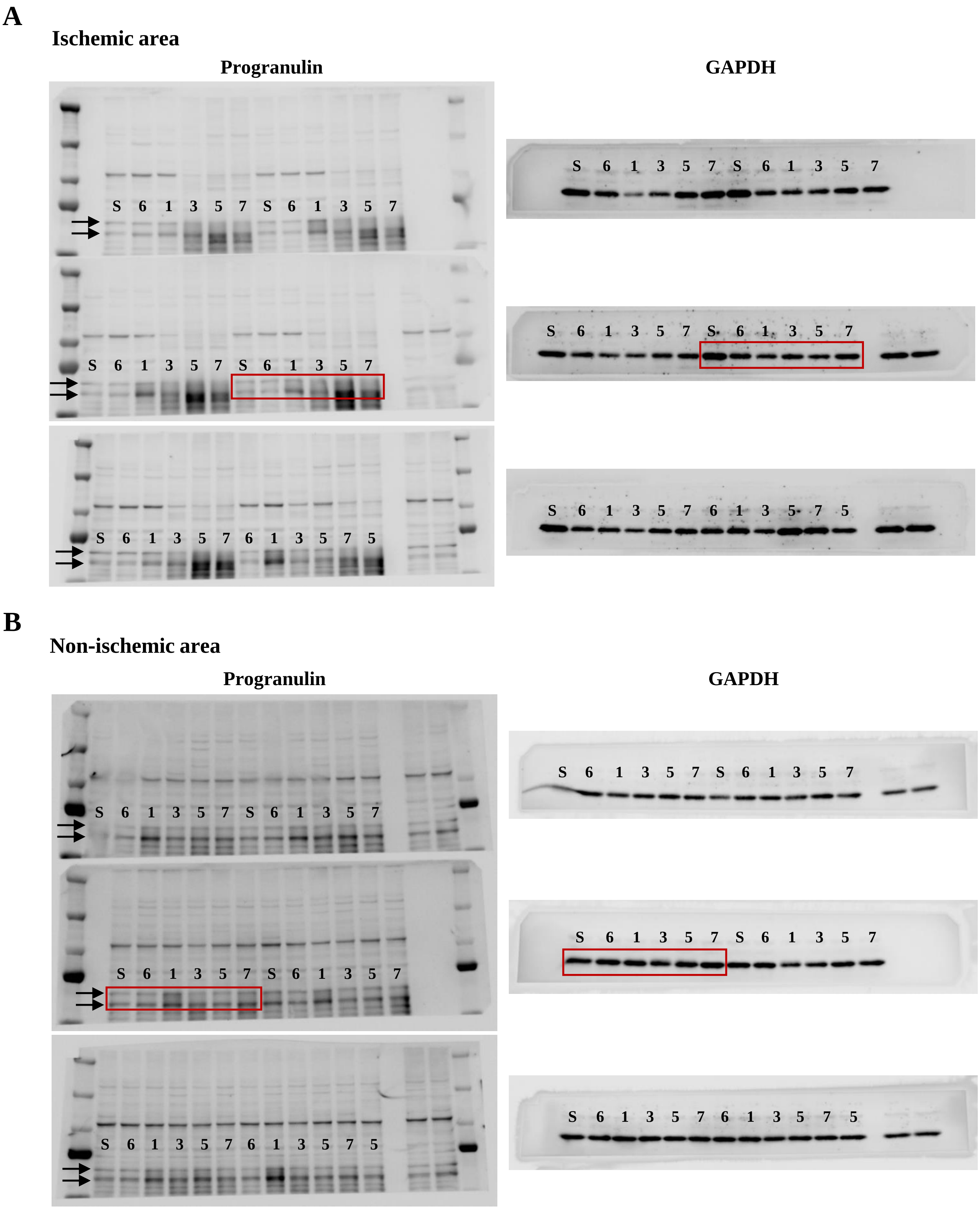

Supplementary Figure 1. The blots of the expression of progranulin protein after occlusion of left coronary artery (LCA) using western blotting.
(A) Immunoblotted images to determine the levels of expression of progranulin protein in ischemic area after occlusion of LCA. (B) Immunoblotted images to determine the levels of expression of progranulin protein in non-ischemic area after occlusion of LCA. Arrows indicate the site of progranulin protein expression (58-68 kDa). The blots in squares are used for the cropped blots in Figure 1. S: Sham; 6: 6 hours after occlusion of LCA; 1: 1 day after occlusion of LCA; 3: 3 days after occlusion of LCA; 5: 5 days after occlusion of LCA; 7: 7 days after occlusion of LCA.

Supplementary Figure 2

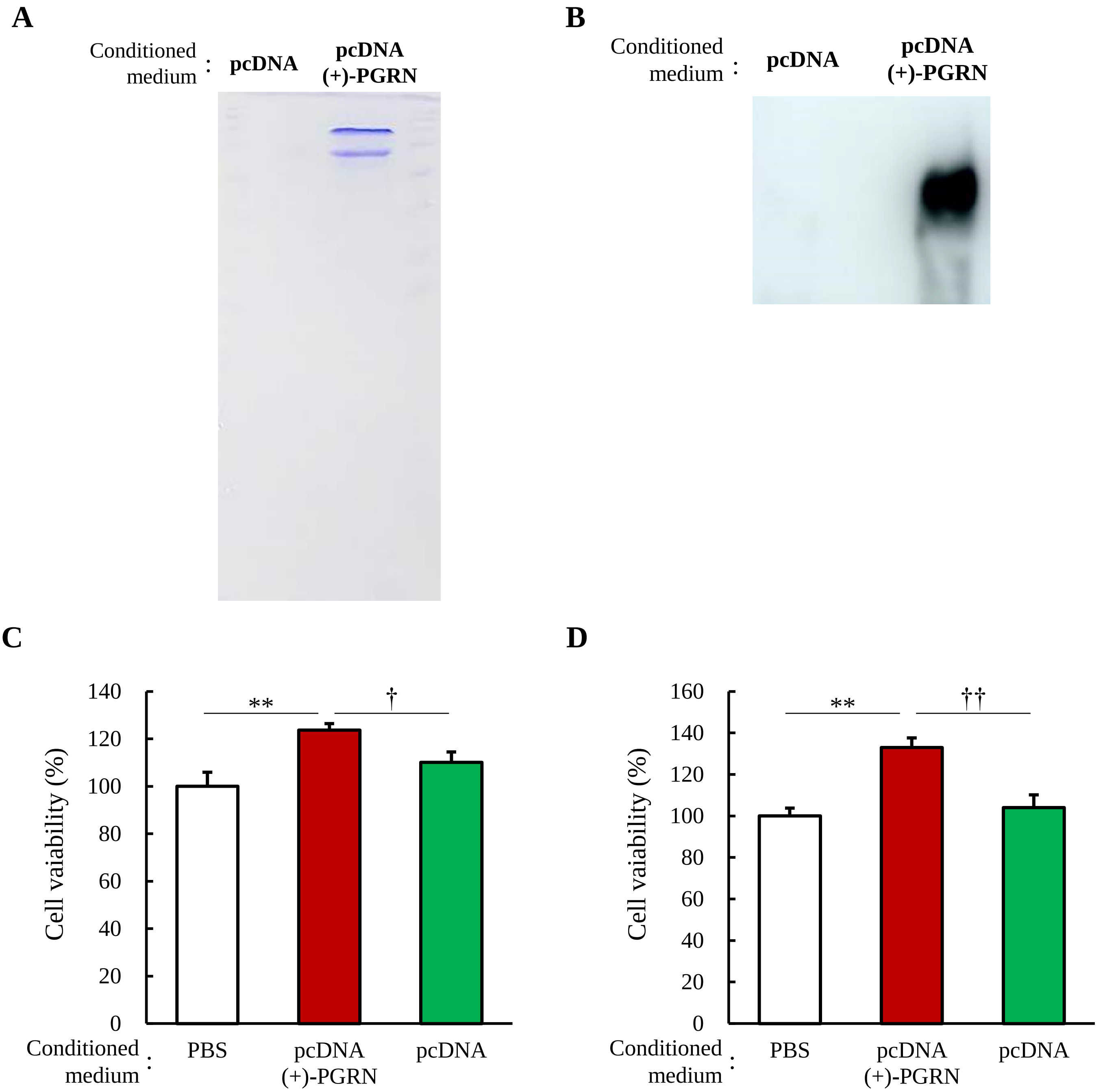

Supplemental Figure 2 Effects of conditioned medium of SH-SY5Y cells transfected with pcDNA3.1 (+)-progranulin.

(A) The images of CBB assay for the conditioned medium of SH-SY5Y transfected with pcDNA3.1 (+)-PGRN and pcDNA3.1. (B) The images show progranulin in the conditioned medium of SH-SY5Y transfected with pcDNA3.1 (+)-PGRN and pcDNA3.1 by western blotting. (C) CCK-8 assay shows the cell viability in H9c2 treated with PBS and the conditioned medium of SH-SY5Y transfected with pcDNA3.1 (+)-PGRN and pcDNA3.1. (D) CCK-8 assay shows the cell viability in SH-SY5Y treated with PBS and the conditioned medium of SH-SY5Y transfected with pcDNA3.1 (+)-PGRN and pcDNA3.1. In the conditioned medium of SH-SY5Y transfected with pcDNA3.1 (+)-PGRN-treated group, cell viability of H9c2 and SH-SY5Y significantly increased compared with the conditioned medium of SH-SY5Y transfected with pcDNA3.1-treated group, and PBS-treated group. It was no significant difference of cell viability in H9c2 and SH-SY5Y between the conditioned medium of SH-SY5Y transfected with pcDNA3.1-treated group, and PBS-treated group. Data are the means ± SEM. (n=6) $^{**}p<0.01$ vs. PBS-treated group, $^{\dagger}p<0.05$, $^{\dagger\dagger}p<0.01$ conditioned medium of SH-SY5Y transfected with pcDNA3.1-treated group (two-tailed Student's *t*-test)

Supplementary Figure 3

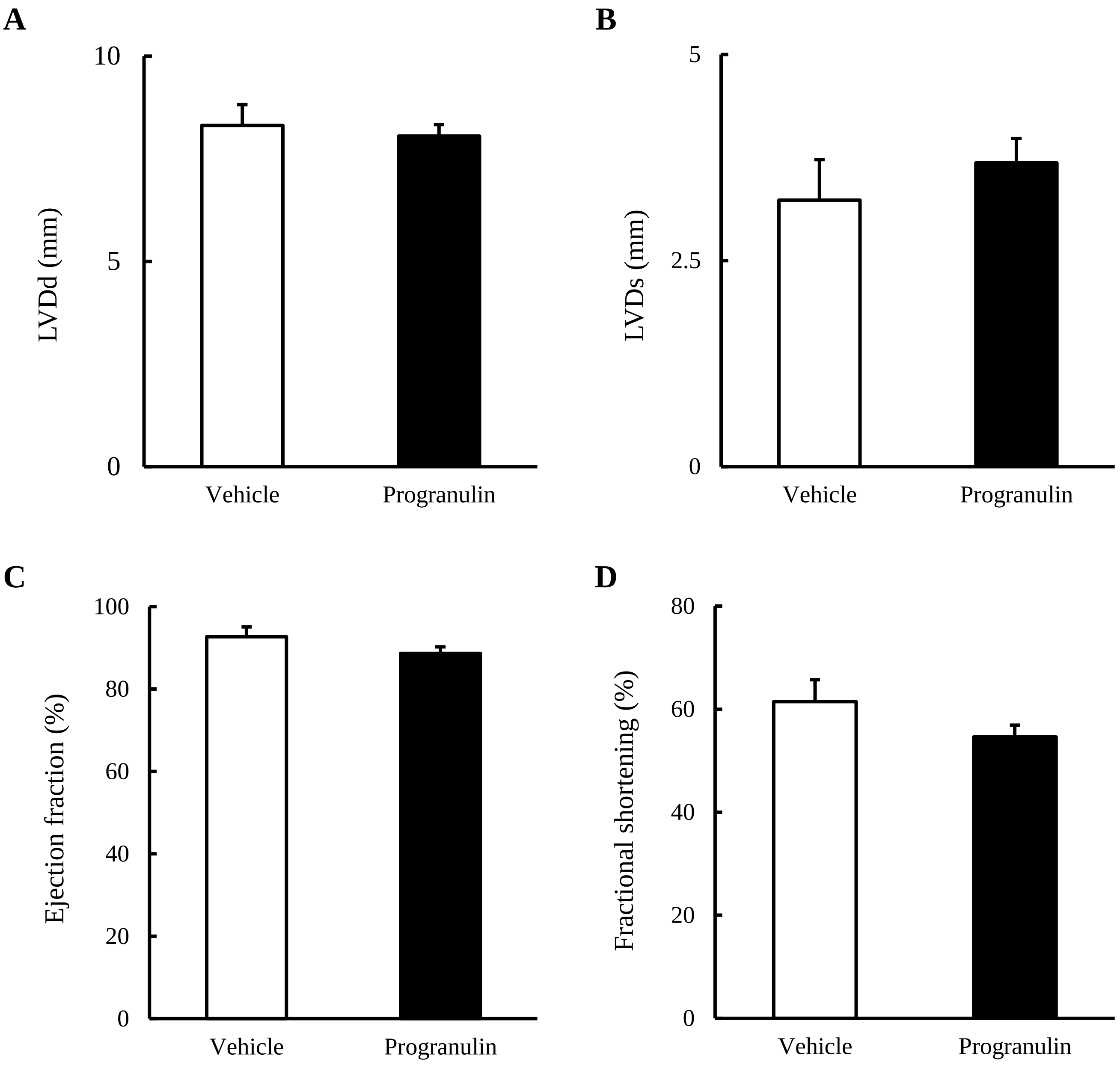

Supplementary Figure 3 The cardiac function before myocardial ischemia-reperfusion injury in the rabbits.

(A) Left ventricular internal diameter at diastole (LVIDd), (B) left ventricular internal diameter at systole (LVIDs), (C) ejection fraction (EF), and (D) fractional shortening (FS) were measured by echocardiography before myocardial ischemia/reperfusion injury. Data are the means ± SEM. (n=3-7) N.S. vs. vehicle-treated group (two-tailed Student's *t*-test)
